# ABT-737 Induces Bim Expression via JNK Signaling Pathway and Its Effect on the Radiation Sensitivity of HeLa Cells

**DOI:** 10.1371/journal.pone.0052483

**Published:** 2012-12-20

**Authors:** Huan Wang, Yue-Bo Yang, Hui-Min Shen, Jian Gu, Tian Li, Xiao-Mao Li

**Affiliations:** Gynecology Department, Third Affiliated Hospital of Sun Yat-sen University, Guangzhou, China; Osaka University Graduate School of Medicine, Japan

## Abstract

ABT-737 is a BH3 mimetic small molecule inhibitor that can effectively inhibit the activity of antiapoptotic Bcl-2 family proteins including Bcl2, Bcl-xL and Bcl-w, and further enhances the effect of apoptosis by activating the proapoptotic proteins (t-Bid, Bad, Bim). In this study, we demonstrate that ABT-737 improved the radiation sensitivity of cervical cancer HeLa cells and thereby provoked cell apoptosis. Our results show that ABT-737 inhibited HeLa cell proliferation and activated JNK and its downstream target c-Jun, which caused the up-regulation of Bim expression. Blockade of JNK/c-Jun signaling pathway resulted in significant down-regulation of ABT-737-induced Bim mRNA and protein expression level. Also, ABT-737 could evoke the Bim promoter activity, and enhance the radiation sensitivity of HeLa cells via JNK/c-Jun and Bim signaling pathway. Our data imply that combination of ABT-737 and conventional radiation therapy might represent a highly effective therapeutic approach for future treatment of cervical cancer.

## Introduction

Cervical cancer is the second most common cancer diagnosed in women besides breast cancer. The cause of it, however, remains in the dark even though several contributing factors have been implicated in the past decades. The current conventional treatment for cervical cancer consists of surgery and radiation therapy. The use of radiation therapy started early this century, and it is now still considered as the pre-eminent treatment for cervical cancer since it can be widely used in different stage of cervical cancer. Hence, seeking a new path to improve the radiation sensitivity of cervical cancer cells will definitely provide the opportunity for achieving better prognosis of cervical cancer in the future.

ABT-737 is a small molecule inhibitor mimicking the function of BH3-only protein (i.e. Bad). It binds to Bcl-xL and Bcl-2 (Ki≤1 nmol/L) with high affinity at their hydrophobic grooves as antagonists, which disrupts the Bcl-2/Bax association and thereby provokes cell apoptosis [1]. Previous studies have shown that ABT-737 not only participated in the apoptotic signaling pathway through inhibiting the function of Bcl-2/Bcl-xL, it could also enhance apoptosis induced by JAK inhibition or JNK activation. [2]–[5]. Moreover, ABT-737 was found to promote the initiation of autophagy. However, the mechanism involved is not yet elucidated.

c-Jun N-terminal kinase (JNK) is a stress activated kinase (SAPK) that belongs to the mitogen-activated protein kinase (MAPK) family. It was first discovered at 1990 for its ability to phosphorylate c-Jun on Ser-63 and Ser-73 within the N-terminal transactivation domain. Activated JNK has a large number of downstream substrates which are mostly nuclear transcription factors (i.e. AP-1 components JunB, JunD, ATF2, ELK-1, DPC4, et). And it was found recently that JNK could phosphorylate cytoplasmic protein such as paxillin, and the mitochondrial membrane proteins such as the Bcl-2 family members Bcl-xl and Bim. The JNK signaling pathway is involved in multiple physiological processes, including embryonic development, immune response and cell differentiation. It is also the key player in different pathological process, especially in tumor progression [6].

Bim (Bcl-2 interacting mediator of cell death), a member of BH3-only subfamily of the Bcl-2 protein family, is an essential proapoptotic protein that plays a key role in maintaining the haematopoietic homeostasis, and acts as a barrier against autoimmunity and cancer development. Bim is widely expressed in different type of cells, and has several isoforms. Bim is localized to mitochondria, smooth endoplasmic reticulum, and perinuclear membranes in cells [1].Upon certain apoptotic stimuli, Bim can initiate apoptosis by directly activating Bax through interaction with the Bcl-2/Bax herterodimer complex, which can further induces mitochondrial cell death. The expression of Bim is highly controlled by its transcriptional and post-translational levels. Activation of JNK signaling pathway has been previously shown to correlate with the expression level of Bim.

The precise role of JNK in ABT-737-induced apoptosis is not entirely clear; however, current evidence suggests that it might involve the interaction with its targeting protein such as c-Jun and the Bcl-2 family proteins. In the present study, we demonstrated that ABT-737 activated c-Jun via JNK signaling pathway in cervical cancer, and thereby regulated the expression of Bim. These results open up the possibility of using ABT-737 as a single agent to improve the radiation sensitivity of cervical cancer cells in the future clinical treatment.

## Results

### 1 ABT-737 Inhibited the HeLa Cell Proliferation

**Figure 1:**
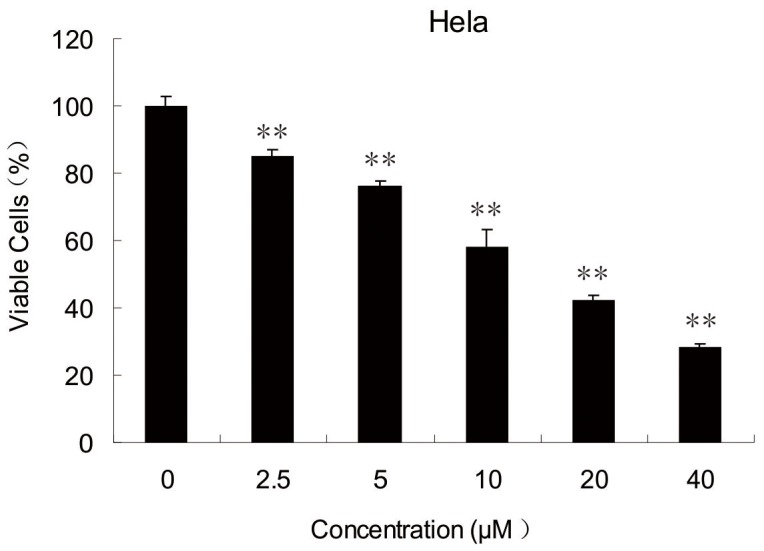
ABT-737 inhibited cell growth in HeLa cells. Cells were treated with 2.5, 5, 10, 20 and 40 µM ABT-737 for 24 h before and the cell viabilities were determined by MTT assay. Error bars represent standard deviations (SD) calculated from three parallel experiments (**, p<0.01 VS Control Group).

The growth effect of ABT-737 on human cancer cervical HeLa cells was evaluated using MTT assay. MTT (3-(4,5-Dimethylthiazol-2-yl)-2,5-diphenyltetrazolium bromide) is a yellow tetrazole which can be reduced to purple formazan dye in living cells. The purple solid product is then solved in dimethyl sulfoxide (DMSO) and the absorbance of resultant purple solution is quantified by a spectrophotometer. MTT assay is frequently employed to accesses the cell viability and determine the cytotoxicity of medicinal agents. Comparing to the control group, HeLa cell proliferation was markedly inhibited in a dose-dependent manner after treatment with 2.5, 5, 10, 20 and 40 µM ABT-737 for 24 h. The result was considered to be significant as P value was less than 0.01 (Fig. 1).

### 2 ABT-737 Activated JNK and its Downstream Target c-Jun

**Figure 2:**
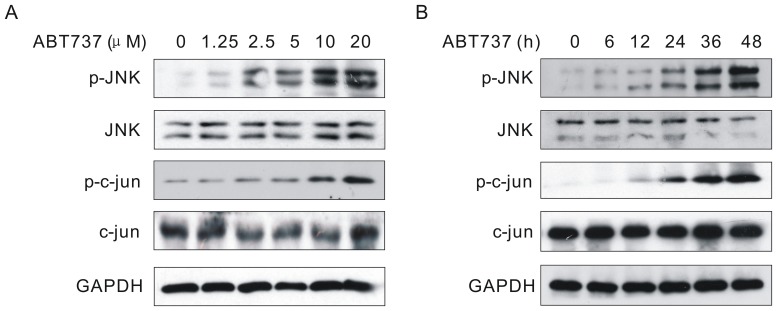
ABT-737 activated JNK and c-Jun. HeLa cells were treated with various concentrations of ABT737 for 24 h (A) or with 10 µM ABT737 for the indicated periods (B). After treatments, cell lysates were analyzed by immunoblotting with JNK, phospho-JNK, c-Jun and phospho-c-Jun antibodies.

As mentioned earlier, the BH-3 mimetic ABT-737 can antagonize the antiapoptotic function of Bcl-2/Bcl-xL complex and provoke apoptosis. Nevertheless, the mechanism involved is not fully understood. Numerous studies have shown that JNK signaling pathway is involved in the regulation of apoptosis. To investigate if JNK signaling pathway was activated in the presence of ABT-737, we first used Western Blotting to examine the phosphorylation activities in the JNK signaling pathway. As shown in Fig. 2, the phosphorylation level of JNK was up-regulated time and dose-dependently in cells treated with ABT-737. Fig. 2A showed that the ABT-737-induced phosphorylation of JNK started at the concentration of 1.25 µM and gradually increased dose-dependently. In Fig. 2B, JNK was continuously activated during the 48 h treatment, with an obvious phosphorylation of JNK starting from 6 h. Moreover, the phosphorylation level of JNK downstream target c-Jun was also elevated accordingly. These results indicated that JNK signaling pathway was continuously activated by ABT-737.

### 3 ABT-737 Induced the Expression of Proapoptotic Protein Bim

**Figure 3:**
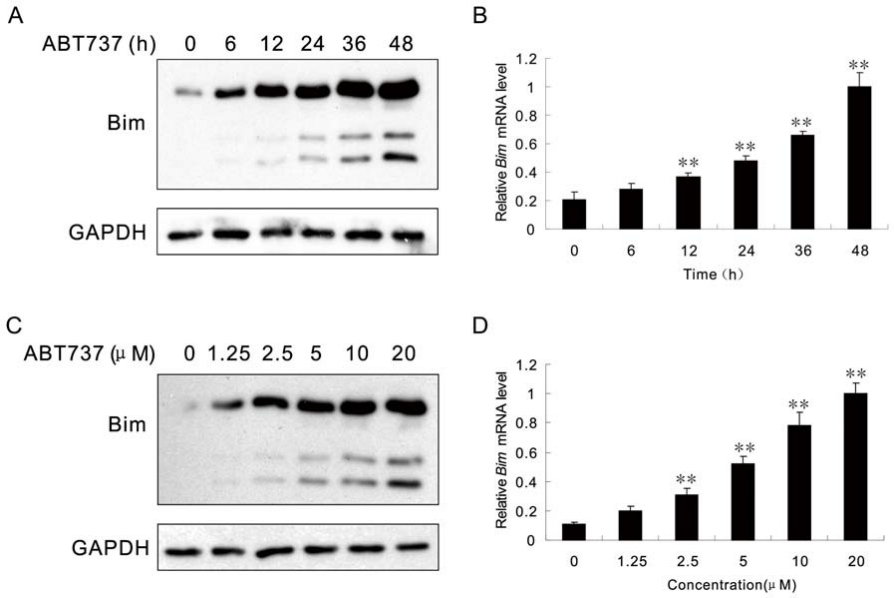
Bim protein and mRNA levels are regulated by ABT-737. HeLa cells were treated with 10 µM ABT737 for the indicated periods. Cell extracts were analyzed by Western Blot using Bim and GAPDH antibodies (A). The expression level of Bim mRNA was detected by Real-time RT-PCR (B). HeLa cells were treated with various concentrations of ABT737 for 24 h. Cell extracts were analyzed by Western Blot using Bim and GAPDH antibodies (C). The expression level of Bim mRNA was detected by Real-time RT-PCR (D). Error bars represent standard deviations (SD) calculated from three parallel experiments (**, p<0.01 VS Control Group).

As ABT-737 can trigger apoptosis via inhibiting the Bcl-2/Bcl-xL complex and Bim is the proapoptotic protein, we were interested to find out if ABT-737 could also affect the expression of Bim. As indicated in Fig. 3, the expression level of Bim in HeLa cells treated with ABT-737 was up-regulated in a –dose and time-dependent manner. Western Blot analysis results showed that in cells treated with 10 µM ABT-737, Bim expression was markedly increased after 24 h (Fig. 3A). The mRNA level of Bim was also up-regulated time-dependently (Fig. 3B). For HeLa cells treated with various concentration of ABT-737 for 24 h, the Bim expression was elevated dose-dependently and exhibited an obvious increase when the ABT-737 concentration reached 5 µM (Fig. 3C). Same effect was also observed for the corresponding Bim mRNA level (Fig. 3D). These results suggested that ABT-737 not only up-regulated the JNK expression but also the expression of Bim.

### 4 Inhibition of JNK Suppressed the ABT-737-induced Bim Expression

**Figure 4:**
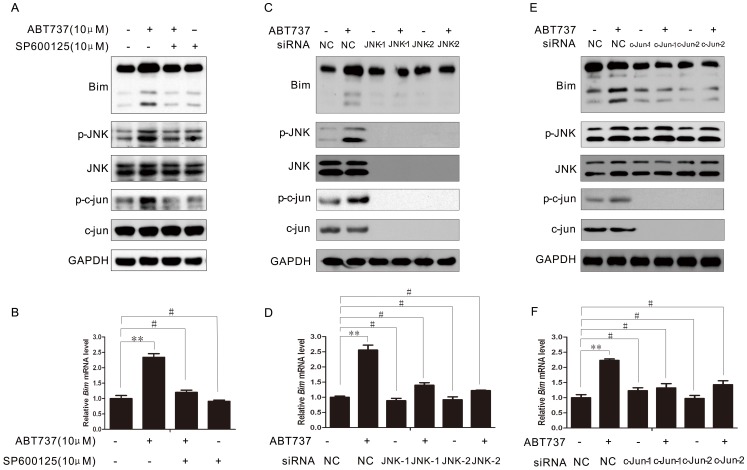
Inhibition of JNK suppressed the ABT-737-induced Bim expression. HeLa cells were treated with ABT737 for 24 h, in the absence or presence of 10 µM SP600125. Then cells were lysed and the indicated proteins were analyzed by Western Blotting (A). Real-time RT-PCR showed Bim mRNA expression in HeLa cells was blocked by SP600125 (B). HeLa cells were transfected with negative control or JNK siRNA for 48 h, then treated with or without ABT737 for 24 h. The cells were lysed and the indicated proteins were analyzed by Western Blotting (C). Real-time showed that silencing of JNK by siRNA blocked ABT737-induced Bim mRNA expression in HeLa cells (D). HeLa cells were transfected with negative control or c-jun siRNA for 48 h, then treated with/without ABT737 for 24 h. The cells were lysed and the indicated proteins were analyzed by Western Blotting (E). Real-time showed that silencing of c-jun by siRNA blocked ABT737-induced Bim mRNA expression in HeLa cells (F). Error bars represent standard deviations (SD) calculated from three parallel experiments (**, p<0.01; #, p>0.05).

To determine of the expression of Bim was required for the activation of JNK signaling pathway, we first simultaneously treat HeLa cells with JNK small molecule inhibitor SP600125 and ABT-737. SP600125 is a reversible ATP-competitive inhibitor that can compete with protein substrates for the binding site and thereby effectively inhibit the function of JNK. For the combination group, HeLa cells were first treated with 10 µM SP600125 for 1 h, followed by treatment with 10 µM ABT-737 for 24 h. Western Blot analysis showed that expression of Bim in cells received the combination treatment was slightly decreased compared with that in cells treated with only ABT-737. The phosphorylation level of JNK and c-Jun were also decreased as expected (Fig. 4A). The decrease in Bim mRNA levels also confirmed the suppressive effect when JNK was inhibited (Fig. 4B).

To rule out the non-specific effect of the JNK inhibitor, we further blocked the JNK signaling pathway with JNK specific siRNA instead to demonstrate the suppressive effect on Bim expression when the function of JNK was inhibited. We designed and validated two different siRNA against JNK. Immunoblotting results indicated that Bim expression level was decreased as we expected after the JNK knocked-down. This inhibitory effect was also observed in the ABT-737-treated cells (Fig. 4C). Notably, Down-regulation of the phosphorylation level of JNK inhibited the activity of c-Jun and its transcription, which further affect its protein level. This finding is consistent with previous studies [7]. Again, the RT-PCR results on the Bim mRNA expression were consistent with the Immunoblotting results (Fig. 4D). The Bim mRNA level in HeLa cells with suppressed JNK function further confirmed that the expression of Bim was compromised, and this effect could not be reversed by treatment with ABT-737.

The role of the novel downstream effector c-Jun in ABT-737-induced Bim expression was essential. Its phosphorylation level was found to be up-regulated in ABT-737 treated cells, which implied that the transcription level of c-Jun might be the key regulator in JNK-mediated Bim expression. To investigate the role of c-Jun in ABT-737 treated cells, we first knocked-down c-Jun gene using two c-Jun specific siRNA. The fact that not only the expression of c-Jun but also the p-c-Jun level were decreased indicating that the function of c-Jun as a transcription factor was compromised (Fig. 4E). As we expected, the Bim protein expression was dramatically decreased as well as its mRNA level, which suggested that suppression of the function of c-Jun resulted in down-regulation of Bim expression and Bim might be the potential downstream target of c-Jun (Fig. 4F).

Based on our results, we suggest that activation of JNK signaling pathway was necessary for the ABT-737-induced Bim expression.

### 5 ABT-737 Evoked the Activation of Bim Promoter

**Figure 5:**
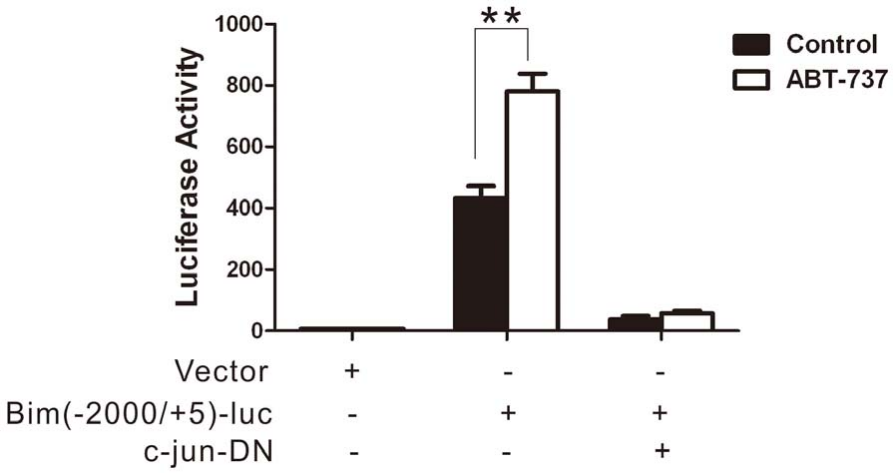
ABT737 evoked the activation of Bim promoter. HeLa cells were transfected with the indicated plasmids. PCMV-RL was co-transfected as internal control. 16 hours after transfection, the cells were treated with or without 10 µM ABT737 for 24 h. Luciferase activities were detected. Error bars represent standard deviations (SD) calculated from three parallel experiments (**, p<0.01).

To investigate if ABT-737 evoked Bim promoter activation via c-Jun, reporter gene assay was employed. The Bim promoter full length reporter Bim (−2000/+5)-luc was constructed and transfected into HeLa cells. 16 h after transfection, cells were treated with 10 µM ABT-737 for 24 h and the luciferase reporter activity was evaluated. The result showed that Bim (−2000/+5)-luc promoter activation was strongly evoked by ABT-737. To further study, we constructed the plasmid expressing c-jun-DN, which the activity of c-jun was inhibited. HeLa cells were co-transfected with Bim (−2000+5)-luc and c-jun-DN. After 16 h transfection, cells were incubated in the presence of ABT-737 or not. The data showed that inhibition of c-jun abolished the activation of Bim promoter. (Fig. 5).

### 6 ABT-737 Induced Apoptosis via JNK/c-jun Pathway

**Figure 6:**
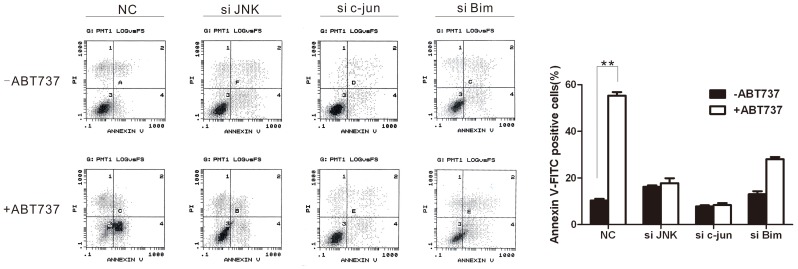
ABT-737 induced apoptosis via JNK/c-jun pathway. HeLa cells transfected with siRNA targeting JNK, c-jun or Bim were incubate in the presence of 10 µM ABT-737 for 24 h or not, then stained with Annexin V-FITC. Flow cytometry analysis was performed to measure the percentage of apoptosis cells. Error bars represent standard deviations (SD) calculated from three parallel experiments (**, p<0.01).

When a cell undergoes apoptosis, the membrane phospholipid phosphatidylserine (PS) is translocated from the inner leaflet of the phospholipid bilayer to the cell surface. Annexin V when labeled with a fluorescent tag, such as FITC, can be used with flow cytometry to measure this event. To examine whether ABT-737 induced-apoptosis is via JNK/c-jun pathway, the HeLa cells was transfected with siRNA targeting JNK or c-jun, and then incubated with 10 µM ABT-737 for 24 h. Annexin V staining was used to identify apoptotic cells (Annexin V-FITC positive). As shown in Fig. 6, ABT-737 induced cell apoptosis; while apoptosis decreased in cells treated with siRNA targeting JNK or c-jun regardless of the presence of ABT-737, indicating ABT-737 induced apoptosis via JNK/c-jun pathway. When cells transfected with siRNA targeting Bim were treated with ABT-737, although the percentage of apoptosis cells was compromised comparing NC group, apoptosis still happened. It was reported that ABT-737 functionally mimics action of BH3 proteins (i.e., Bim), which can overcome and induce apoptosis in Bim knockdown hematopoietic cells [3]. Our data showed that JNK induced apoptosis via Bim but not only Bim. There maybe other pathway involved in JNK-mediated apotosis, for example, TNF-α or caspase-8.

### 7 ABT-737 Enhanced Apoptosis Induced by Radiation in HeLa Cells

**Figure 7:**
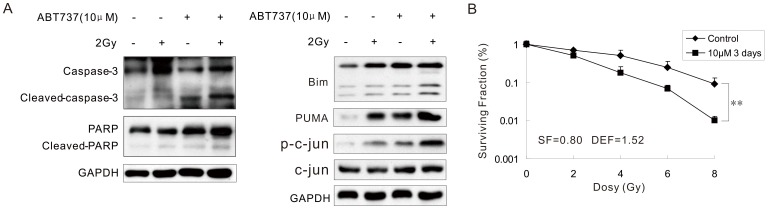
ABT737 enhanced apoptosis induced by radiation in HeLa cells. Western blot analysis of the indicated proteins in HeLa cell treated with 10 µM ABT737 and/or 2Gy radiation for 24 h (A). The survival fractions of cells treated with radiation (0, 2, 4, 6 and 8 Gy) and 10 µM ABT-737 for 3 days were determined by colony formation assay (B). Error bars represent standard deviations (SD) calculated from three parallel experiments (**, p<0.01).

To investigate the effect of ABT-737 on the radiation sensitivity of in JNK/c-Jun activated HeLa cells, we treated HeLa cells with both radiation (2 Gy) and 10 µM ABT-737 and detected the expression of cleaved-Caspase-3 and cleaved-Parp, which were two essential proteins involved in the apoptosis pathway. Fig. 7A(left) clearly showed that, cleaved-Caspase-3 and cleaved-Parp were observed in cells treated with ABT-737, indicating ABT-737-induced apoptosis. In addition, the expression levels of both cleaved-Caspase-3 and cleaved-Parp were increased in the combination group comparing to those in the groups that were treated with only ABT-737 or radiation. It suggested that the apoptosis induced by radiation was enhanced by simultaneous treatment with ABT-737. At the same time, Bim and PUMA, two pro-apoptotic proteins of bcl-2 family, have been examined. As shown in Fig 7A (Right), both ABT-737 and conventional radiation could enhance these two proteins’ expression levels, and their expression were much higher in the combination group.

Result of the colony formation assay indicated that the clonogenic survival of HeLa cells was decreased after combining the radiation (0, 2, 4, 6 and 8 Gy) with treatment of 10 µM ABT-737 for 3 days. The DEF value s for the combination group was 1.52. The Survival Fraction (SF) value is 0.80 (Fig. 7B).

## Discussion

Programmed cell death is any form of cell suicide triggered by various physiological stimuli and mediated by intracellular programs. It is a fundamental biological process for tissue development and renewal during an organism’s life cycle. Overall, mammalian cell death can be divided into three types morphologically: apoptosis, autophagic cell death (ACD) and necrosis. The concept of apoptosis was first brought up by Professor Kerr at 1972 based on morphological criteria. According to him, apoptosis is a distinctive morphological event which is essential in maintaining cellular homeostasis. This highly conserved biological process is initiated by different environmental stimuli and tightly regulated by multiple genes involved in different cell signaling pathways [8]. As more research has been conducted in this filed for the past decades, the apoptosis pathway is well-studied and divided into three phases: the decision phase, the commitment phase and finally the execution phase [9]. The key morphological characteristics occurred during cell apoptosis include membrane blebbing, cell shrinkage, chromatin condensation and intact cellular organelles.

ABT-737 is a small molecule inhibitor developed by the US healthcare company Abbott [10] and currently at the early clinical trial phase [11]. This Bcl-2 family inhibitor was found by using the NMR-based screening technique, parallel synthesis and structure-based design. Fluorescence Polarization (FP) assay showed that ABT-737 binded with high affinity to Bcl-xL, Bcl-2 and Bcl-w (Ki≤1 nmol/L), while it had low affinity towards Mcl-1 (Ki = 0.46±0.11), Bcl-B and A1 (Ki≥1 µmol/L). Early clinical trials revealed that ABT-737, as a single agent, not only could kill certain cancer cells including lymphoma cancer cells [12] and small-cell lung cancer cells [13], but also could improve their radiation sensitivity and chemosensitivity [14], [15]. A recent study found that ABT-737 could enhanced the therapeutic effect of the conventional treatment for pediatric acute lymphoblastic leukemia and at the same time reduce the cytotoxic effect of the treatment [16]. To investigate the molecular mechanism involved in the ABT-737-induced apoptosis, we used human cervical cancer HeLa cells for this study. In the first part of our study, we demonstrated that ABT-737 significantly inhibited the HeLa cell proliferation in a dose-dependent manner.

It has been previously shown that ABT-737 could induce apoptotic cell death. The mechanism involved, however, is not fully understood. JNK can be activated by different type of extracellular signals and cellular stress, such as heat shock, tumor necrosis factor, radiation, ischemic and hypoxic stress [17]. It plays a key role in cellular stress-induced apoptosis [18]. Numerous studies suggested that JNK could inhibit cell proliferation through activating its downstream effecter c-Jun [19]. Our data demonstrated that both phosphorylated JNK and c-Jun were time and dose-dependently activated in cells treated with ABT-737, indicating the effective activation of JNK signaling pathway. Howard AN, et al have reported ABT-737 could induce oxidative stress [20]. And ROS/JNK pathway is widely reported. Therefore, we thought that the activation of JNK pathway was just a part of signaling storms triggered by ABT-737. However, mechanisms in which ABT-737 induces JNK activation must be explored.

As reported previously, JNK-induced apoptosis was shown to be mediated by JNK cytoplasmic substrates, such as phosphorylation and activation of the proapoptotic proteins BAX and Bim [21], or regulated through the activated mitochondrial apoptotic pathway, such as phosphorylation and inhibition of the Bcl-2 family proteins including Bcl-2, Bcl-xL and Mcl-1 [22]. Our present data also revealed that in HeLa cells treated with ABT-737, the expression of the BH3 subfamily protein Bim was markedly up-regulated in a time and dose-dependent manner.

To further clarify the correlation between the expression of Bim and the JNK signaling pathway, we first suppressed the function of JNK using a JNK-specific inhibitor SP600125 and found that the Bim expression level was down-regulated when cells were treated with both SP600125 and ABT-737. To further rule out the non-specific effect of the JNK inhibitor, we repeated the experiment with a JNK-specific siRNA. RNA interference (RNAi) is a post-transcriptional process by which a small double-stranded DNA (dsDNA) with ∼20bp nucleotides targets at the homologous messenger RNA (mRNA) and leads to gene silencing. The Western Blotting analysis indicated an effective know-down of JNK gene with this JNK specific siRNA by showing the significant decrease in the expression of both JNK and c-Jun. The inhibitory effect was also observed in the expression of Bim. The RT-PCR results further confirmed the suppression of Bim expression as the mRNA level of Bim was down-regulation, even in the ABT-737-treated cells. All the data points to the fact that ABT-737-induced Bim expression via the JNK signaling transduction pathway in cervical cancer HeLa cells.

Notably, unlike the JNK-induced apoptosis pathway mentioned previously, our data suggested that the phosphorylation of JNK downstream transcription factor c-Jun was highly involved in the regulation of the JNK-induced apoptosis. The phosphorylation level of c-Jun was continuously elevated when JNK was kept activated for 48 h. In order to investigate the possible mechanism behind it, we designed a c-Jun specific siRNA and transfected it into the cells. Consisting with the Western Blotting analysis, RT-PCR results indicated that the mRNA level of Bim was decreased after the knocked-down of c-Jun, suggesting that protein Bim might be the downstream target of c-Jun.

C-Jun is one of the essential transcription factors in the JNK signaling pathway, and it can induce the expression of its downstream substrate. Hence, the possible mechanism involved in the ABT-737-induced apoptosis was likely that activated c-Jun was first translocated into the nucleus and then mediated its downstream target Bim expression at the transcriptional level. To test this hypothesis, we first obtained the genetic information of the Bim promoter using bioinformatic technique to locate the conserved binding sequence for the reporter genes. We then performed the luciferase reporter assay to examine the activity of the Bim promoter. Our results revealed that the activation of Bim (−2000/+5) promoter was drastically evoked in the cells treated with 10 µM ABT-737 for 24 h, which consisted with our hypothesis. Meanwhile, inhibition of JNK/c-JUN attenuated the Bim luciferase activity.

There is evidence showing that c-Jun and GADD153 can promote cell apoptosis when the JNK signaling pathway is activated [23], [24]. Also in cerebellar granule neurons, withdrawal of survival signal will trigger the phosphorylation of c-Jun and further induce cell apoptosis [25]. For the mitochondrial apoptosis pathway, the release of cytochrome C and change in the mitochondrial membrane potential are the two key upstream factors. The proapoptotic stimuli trigger the release of cytochrome C from the mitochondria and the “free” cytochrome C will form a complex with Apaf1. The Apaf1/cytochrome C complex then recruits Pro-Caspase-9 and cleaves it. Activated Caspase-9 further triggers the cleavage of Caspase-3 and Parp, thereby initiates apoptosis [26]. As Caspase-3 and Parp are two fundamental proteins in the apoptosis pathway, we therefore examined the expression of cleaved-Caspase-3 and cleaved-Parp in the ABT-737 treated HeLa cells to further demonstrate that ABT-737 could induce apoptosis in JNK/c-Jun activated cells, which is consistent with the result of Annexin V staining. In addition, JNK/c-jun knocked-down decreased ABT-737-induced apoptosis, indicating ABT-737 induced HeLa apoptosis via JNK/c-jun/Bim pathway. Bim eventually activates pro-apoptotic Bcl-2 family members (BAX or BAK) to induce apoptosis. There are already two suggested mechanisms for BAX/BAK activation by Bim: 1) Bim directly binds to and activation BAX; and/or 2) Bim binds to anti-apoptotic Bcl-2 family proteins such as Bcl-2 so that Bcl-2 cannot inhibit BAX anymore, thereby inducing BAX activation [27]. However, this hypothesis requires more empirical evidence in our study.

Radiation therapy is the pre-eminent treatment for cervical cancer. Our data point toward the notion that, when combining the radiation therapy with ABT-737 treatment, the clonogenic survival of human cervical cancer HeLa cells were effectively decreased and cell apoptosis was significantly increased. In conclusion, ABT-737 could induce the Bim expression via activation of JNK/c-Jun signaling pathway and thus increased the radiation sensitivity of HeLa cells. These findings will shed the light on the future clinical treatment for human cervical cancer as well as gain more insights in small molecule therapy.

## Materials and Methods

### 1 Ethics Statement

This research has been approved by the Ethics Committee of The Third Affiliated Hospital of Sun Yat-sen University.

### 2 Drugs and Reagents

Fetal bovine serum (FBS) was purchased from Gibco® (New York, USA). 3-(4,5-dime-thylthiazol-2-thiazolyl)-2,5-diphenyltetrazolium bromide (MTT) and DMSO were obtained from Sigma-Aldrich (St. Louis, USA). Antibodies for Bim, JNK and p-JNK were acquired from Cell Signaling Technology (New York, USA). Antibodies for c-Jun, p-c-Jun, Caspase-3, Parp, GAPDH and HRP secondary antibody were products of Santa Cruz Biotechnology (Santa Cruz, USA). ABT-737 was from Biochempartner (Shanghai, China).

### 3 Cell culture

Human cervical cancer cells were cultivated in DMEM supplemented with 10% FBS, penicillin (50 U/ml) and streptomycin (50 µg/ml) at 37°C in a humidified atmosphere of a 5% (v/v) CO_2_ in air. All experiments were carried out with cells at the logarithmic growth phase.

### 4 MTT Assay

Cells were seeded in a 96-well plate with a density of 5000 cells per well in 200 µL DMEM. Cells were either treated with various concentration of ABT-737 or with 0.1% DMSO then incubated at 37°C in 5% CO_2_ for 72 h. 10 µL of 5 mg/mL MTT was then added into each well and the plate were incubated at 37°C in 5% CO2 for another 4 h. After complete removal of the medium, 100 µL of DMSO was added into each well to dissolve the insoluble purple formazan product. The absorbance of the resultant purple solution was quantified by a spectrophotometer. The 50% inhibitory rates (%) were calculated according to the Bliss method: Inhibitory rate = (1-the average OD value of treatment group/the average OD value of the control group) ×100%.

### 5 Colony Formation Assay

Cells were first treated with different dose of radiation (X-ray Model F34-I), then cultivated in 6-well plates containing different concentration of ABT-737 at 37°C in 5% CO_2_. After 6–8 days, cells were fixed in anhydrous ethanol and stained with crystal violet and colonies were counted. The survival fraction was calculated based on the plating efficiency and clonogenic survival.

### 6 Western Blot Analysis

Cells were harvested and washed twice with PBS and lyzed in 100µL lysis buffer. The cell lysates were centrifuged at 14,000 g for 15 min at 4°C and the concentrations of protein were determined using the Bio-Rad protein assay (Bio-Rad laboratories). SDS-PAGE sample buffer was added to cell lysate before heated at 95°C for 5 min, and cell lysate containing 40µg protein was loaded in each well of the SDS-PAGE gel. Resolved proteins were electrophoretically transferred to PVDF membrane and incubated sequentially with primary antibodies and horseradish peroxidase–conjugated secondary antibodies. After washing 3 times with TBST solution (10 mmol/L Tris-HCl, PH7.4, 150 mmol/L NaCl, 0.1% Tween20), the protein complex was detected using an ECL chemiluminescence reagent and XAR film (Kodak, New York, USA) as described by the manufactures.

### 7 siRNA Transfection

The target sequence for JNK-specific siRNA was:

1: 5′-GAAAGAATGTCCTACCTTC-3′;

2: 5′-AAAAAGAAUGUCCUACCUUCU-3′ (GeneBank accession number NM002750.2)

The target sequence for c-Jun-specific siRNA was:

1: 5′-UCAUCUGUCACGUUCUUGG-3′;

2: 5′-AGAUGGAAACGACCUUCUATT-3′ (GeneBank accession number NM002228.3).

The target sequence for Bim-specific siRNA was product of Santa Cruz Biotechnology (Santa Cruz, USA).

These siRNA as well as the control siRNA (no silencing) was synthesized by Genema (Shanghai, China). One day before transfection, cells were plated in six-well plates with antibiotic-free growth medium at a density of 1.5×105 cells per well. When cells grew to a confluency of 30–50% on the second day, transfection was performed by using Opti-MEM media (Gibco®, New York, USA), lipofectamine 2000 (Invitrogen, New York, USA) and the siRNA according to manufacturer’s recommendations. After 6 h, the media was replaced with antibiotic-free growth medium.

### 8 RNA Extraction and RT-PCR

Total RNA was isolated using 1 mL Trizol™ according to the manufacturer’s recommendations. The RT-PCR were performed was performed using the ABI PRISM 7900HT sequence detection system in a reaction volume of 25 µL. The primers for Bim and GAPDH were obtained from SybGREEN qPCR primer pairs (OriGene Technologies).

### 9 Luciferase Reporter Assay

Cells were first lyzed in 1xPLB (Passive Lysis Buffer) and centrifuged at 13,000 rpm for 30 s. After complete removal of the supernatant, 100 µL Luciferase Assay Reagent II was added. Dual-luciferase reporter assay system (Promega) was used to detect luciferase activity in HeLa cells according to the manufacturer’s recommendations. A *Renilla* luciferase vector (Promega) was co-transfected to monitor transfection efficiency. After the values of the *Photinus pyralis* firefly activity were recorded, 100 µL of termination reagent (Stop & Glo Reagent) was added to the samples. All luciferase results are reported as relative light units (RLU): the average of the *Photinus pyralis* firefly activity observed divided by the average of the activity recorded from *Renilla* luciferase vector.

### 10 Annexin V-FITC/PI Staining

Cells treated as described previously were collected in 1 ml PBS, re-suspended in binding buffer containing Annexin V-FITC at room temperature for 10 min. Then cells were incubated with PI on ice for 10 min and analyzed by flow cytometry within 1 hour.

### 11 Statistics

Data analysis was carried out using a 2-tailed Student’s t-test with pooled variance. Data are expressed as mean ± SD of at least three sample replicates, unless stated otherwise. In the Figures, *denoted P<0.05, denoted **P<0.01, # denoted P>0.05.
